# Exposure to a Highly Caloric Palatable Diet during the Perinatal Period Affects the Expression of the Endogenous Cannabinoid System in the Brain, Liver and Adipose Tissue of Adult Rat Offspring

**DOI:** 10.1371/journal.pone.0165432

**Published:** 2016-11-02

**Authors:** María Teresa Ramírez-López, Raquel Arco, Juan Decara, Mariam Vázquez, Rosario Noemí Blanco, Francisco Alén, Juan Suárez, Raquel Gómez de Heras, Fernando Rodríguez de Fonseca

**Affiliations:** 1 Departamento de Psicobiología, Facultad de Psicología, Universidad Complutense de Madrid, Campus de Somosaguas s/n, 28223 Pozuelo de Alarcón, Madrid, Spain; 2 Instituto de Investigación Biomédica de Málaga (IBIMA), Unidad de Gestión Clínica de Salud Mental, Hospital Regional Universitario de Málaga, Universidad de Málaga, 29010, Málaga, Spain; Universidade de Santiago de Compostela, SPAIN

## Abstract

Recent studies have linked gestational exposure to highly caloric diets with a disrupted endogenous cannabinoid system (ECS). In the present study, we have extended these studies by analyzing the impact of the exposure to a palatable diet during gestation and lactation on a) the adult expression of endocannabinoid-related behaviors, b) the metabolic profile of adult offspring and c) the mRNA expression of the signaling machinery of the ECS in the hypothalamus, the liver and the adipose tissue of adult offspring of both sexes. Exposure to a palatable diet resulted in a) sex-dimorphic and perinatal diet specific feeding behaviors, including the differential response to the inhibitory effects of the cannabinoid receptor inverse agonist AM251, b) features of metabolic syndrome including increased adiposity, hyperleptinemia, hypertriglyceridemia and hypercholesterolemia and c) tissue and sex-specific changes in the expression of both CB1 and CB2 receptors and in that of the endocannabinoid-degrading enzymes FAAH and MAGL, being the adipose tissue the most affected organ analyzed. Since the effects were observed in adult animals that were weaned while consuming a normal diet, the present results indicate that the ECS is one of the targets of maternal programming of the offspring energy expenditure. These results clearly indicate that the maternal diet has long-term effects on the development of pups through multiple alterations of signaling homeostatic pathways that include the ECS. The potential relevance of these alterations for the current obesity epidemic is discussed.

## Introduction

Obesity and metabolic syndrome prevalence are increasing worldwide [1,2]. Although lifestyle factors contribute to this epidemic, it is becoming clear that nutritional conditions during critical windows of development, including the perinatal period, could impact the future health of offspring and increase the risk of metabolic diseases [3–5]. This phenomenon has been defined as nutritional programming [6].

Furthermore, it is well-known that maternal and postnatal nutrition tends to be excessive in Western societies. Based on this consideration, human and animal studies have focused on the effect of overnutrition during the perinatal period and the disease susceptibility of offspring later in life [7–12]. Specifically, maternal exposure to hypercaloric diets has been linked to the development of features of metabolic syndrome in offspring such as increased adiposity, glucose, insulin and lipid metabolism alterations or higher blood pressure [8,10,11,13]. Additionally, sexual dimorphism has been documented after maternal exposure to inadequate diets. Thus, a maternal high-fat diet or perinatal exposure to junk food modifies the metabolic outcomes and the gene expression profile of several metabolism-related genes in a sex-specific manner [12–14].

On the other hand, food preferences have been found to be altered after maternal exposure to specific diets as well. For instance, it has been reported that exposure to junk food during pregnancy, especially when the mother actively chooses this type of food, increases the preference for this type of food later in life [15,16]. This fact might help aggravate the health outcomes previously documented. Indeed, it has been shown that the metabolic phenotype reported after being exposed to an adverse nutritional environment during the perinatal period seems to be exacerbated when inadequate nutritional conditions carry over to the postweaning period [8,9,13].

Several mechanisms have been identified as key factors in this process of malprogramming. They include leptin dysregulation [17], epigenetic phenomenons [18], alterations in the hypothalamic neuropeptides [19] or modifications in opioid and dopamine signaling of the central reward system [16,18]. Additional mechanisms might contribute to these mediators. Among them is the endocannabinoid system (ECS), which keeps important modulatory relations with the above cited homeostatic mechanisms involved in feeding and metabolic homeostasis [20,21]. However, to date its role in nutritional programming has not been well characterized.

The ECS is a lipid signaling system composed by endogenous ligands (mainly anandamide (AEA) and 2-arachidonoyl glycerol (2-AG), which are direct arachidonic acid/indirect linoleic acid derivatives. The ECS is also comprised of the cannabinoid receptors (CB1 and CB2) and the enzymes required for their synthesis and degradation such as fatty acid amide hydrolase (FAAH), *N*-acyl phosphatidylethanolamine phospholipase D (NAPE-PLD), diacylglycerol lipases (DAGLα and DAGLβ) and monoacylglycerol lipase (MAGL) [22,23].

The ECS plays a pivotal role in brain development, which is particularly critical in important processes such as cell proliferation, lineage fate decisions, phenotypic acquisition, migration or synaptogenesis [24]. Moreover, the ECS is involved in the modulation of food intake and energy homeostasis through central and peripheral mechanisms [25,26]. Indeed, their receptors are widely expressed in important areas regulating feeding behavior and metabolism which include the hypothalamus, the central reward system, liver or adipose tissue [26,27]. Furthermore, the ECS is implicated in the regulation of food preferences and in the perception of palatability. For instance, the cannabinoids enhance sweet responses in taste receptor cells whereas the CB1 inverse agonist AM251 reduces this response [28]. Additionally, pharmacological CB1 receptor blockade could decrease the motivation to obtain highly palatable food when a free choice between two types of food is made possible [29,30]. Thus, the ECS favors feeding behavior, especially for dense-caloric and palatable foods.

The ECS promotes energy storage, decreases energy expenditure [26] and is sensible to dietary modifications. Its hyperactivation has been associated to obesity and metabolic syndrome [31]. Furthermore, hypercaloric diets have been linked to alterations in the expression and density of several components of the ECS in the brain and peripheral tissues. Thus, access to a highly palatable diet could modify the expression and density of CB1 receptors in specific brain regions [32]. It has been also demonstrated that a chronic high-fat diet exposure alters the levels of endocannabinoids and their metabolic enzymes in the pancreas and adipose tissue [33]. Moreover, dietary fatty acids can modify the levels of endocannabinoids in the brain and peripheral organs [34,35].

Considering the importance of the ECS in food intake, energy balance, neurodevelopment and its role in the pathophysiology of metabolic diseases, it has been proposed that the ECS could play an important role in the process of nutritional programming [24]. Thus, an inadequate maternal diet results in a disruption of endocannabinoid signaling at birth, and these changes appear to have long-lasting consequences [36]. However, it is unknown whether the ECS components could be affected later in life after only being exposed to an inadequate maternal diet during the perinatal period.

Given this background, we aim to study the contribution of the ECS in early life programming. Accordingly, we addressed the effect of maternal exposure to a highly palatable diet in male and female offspring that were weaned on a standard diet. Changes in body weight and food intake over time were monitored after weaning. Metabolic parameters in plasma and adiposity were also studied at adulthood. Additionally, the feeding behavior and food preferences of these animals were evaluated when highly palatable food was available for a short period of time. For the first time, we evaluated the expression of several components of the ECS in metabolically relevant tissues such as the hypothalamus, liver and perirenal adipose tissue in adult animals, which were only exposed to a highly palatable diet during the perinatal period. The expression of lipogenic genes and *Ppars* genes was also measured. We hypothesize that perinatal exposure to an inadequate diet could program feeding behavior, metabolic parameters and affect several components of the ECS at adulthood, even though animals were weaned on a standard chow diet. We also proposed that these alterations could be expressed in a sex-specific manner and may increase the vulnerability to develop metabolic diseases later in life.

## Materials and Methods

### Animals, diets and experimental design

The Animal Ethics Committee of the Complutense University of Madrid approved this study. Additionally, it followed the European Directive 2010/63/EU in accordance with the current Spanish regulations (RD 53/2013 and 178/200) on the protection of animals used for scientific purposes.

The perinatal protocol was carried out as previously reported [36]. Initially, experiments were performed in prepuberal female Wistar rats (Harlan, Barcelona, Spain). They were handled, individually housed, and randomly assigned to control (n = 9) or free-choice diet (n = 11), after three weeks of acclimatization.

Control rats (C) received standard chow *ad libitum* (standard chow SAFE A04, Panlab, Barcelona, Spain). In contrast, free-choice rats (P) were allowed to choose between standard rat chow (standard chow SAFE A04, Panlab, Barcelona, Spain) and a highly palatable food composed of a mixture of chocolates, *ad libitum* as well. The mixture of chocolate food was composed of a homogenous mixture of Mars^®^, Snickers^®^, Bounty^®^ and Milka^®^ in equal proportions as previously described [37,38]. A detailed nutritional composition of both types of food is shown in S1 Table. All female rats kept the same experimental diet during the perinatal period. This period included pregestation (from the beginning of the experiment to eight weeks prior to mating), mating, the gestational period and lactation.

Eight weeks after assigning the type of diet, females were allowed to mate with males of the same strain. Successful mating was confirmed by the presence of a vaginal plug or spermatozoa in the vaginal smear the following morning. The day of birth was defined as postnatal day 0 (PN day 0), pups were weighed, sexed and litters were culled to 8 pups, consisting of 4 males and 4 females. During the lactation period, the weight of every pup was recorded three days per week.

At PN day 22–23, offspring from both animal groups were weaned and exposed to standard chow diet (male offspring: n = 15 and n = 17 for control (CC) and free-choice (PC) perinatal diet groups, respectively; female offspring: n = 18 and n = 23 for control (CC) and free-choice (PC) perinatal groups respectively). Rats from the same sex, liter and perinatal diet group were housed together (2–3 rats/cage) where possible. In order to minimize the estrous cycle-related variability, the female rats were closely housed in the same and adjacent cages, and randomly distributed among the different experimental groups [39–41]. Moreover, males were housed in a separate room.

**Fig 1:**
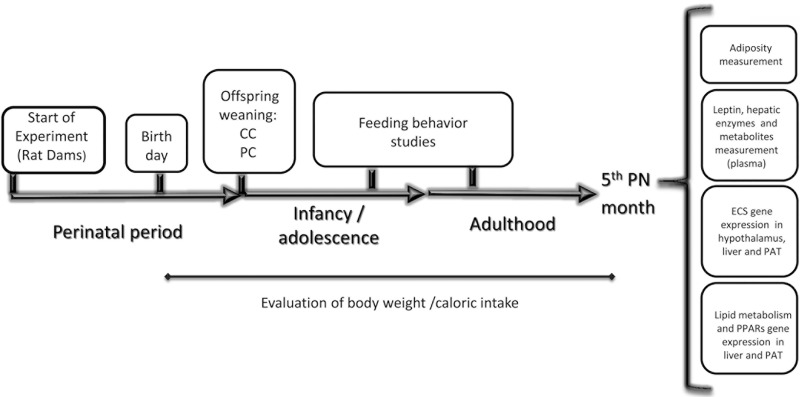
Experimental design. Offspring from rat dams assigned to the experimental diets were studied during different stages of development. After weaning (PN day 22–23), all offspring were exposed to a standard chow diet. Feeding behavior studies (Compulsive Feeding Test and AM251 test) were performed during adolescence and/or adulthood. At the 5^th^ PN month, offspring were sacrificed and adiposity was assessed. Leptinemia and plasma metabolites were also studied. Additionally, the characterization of the ECS, lipid metabolism and PPARs gene expression was evaluated in metabolically-relevant tissues (hypothalamus, liver and PAT). The evolution of body weight and caloric intake was assessed until adulthood (5^th^ PN month).

During the post-weaning period, weight and food intake were measured weekly. Behavioral studies to evaluate food intake and food preferences were performed in adolescence and adulthood. At the 5^th^ postnatal month, three quarters of the male offspring were sacrificed. Then, the perirenal and perigonadal fat were removed to estimate adiposity. Additionally, the whole brain, plasma and samples from perirenal fat and liver were collected for further analysis (Fig 1). To avoid litter effects, samples from at least three litters per perinatal group were used in all determinations.

All animals were maintained at a 12h light-dark cycle with temperature of 22±1°C and were given free access to food and water. The criteria described by Vickers were used to refer to different age stages [42].

### Evaluation of caloric intake and chocolate preference

Food intake was determined by subtracting the amount of each food type left in the cage from the total amount of food provided. To calculate individual food intake when animals were housed in groups, total food intake from each cage was measured and equally divided according to the number of pups per cage. Comparisons among groups were performed by calculating cumulative caloric intake (Kcal/Kg) as well as absolute body weight in each period of the study.

In rat dams assigned to a free choice diet, the chocolate preference during the perinatal period was evaluated. Chocolate preference was calculated as the percentage of chocolate intake over total food provided. The average of chocolate preference in pre-gestation (three weeks prior to mating), gestation (21 days) and lactation (22 days) was recorded and used to compare the chocolate preference between the different perinatal periods.

### Feeding behavior studies

#### Compulsive feeding test

An adaptation of the Compulsive Feeding Test described by Heyne et al. [38] was adopted to assess the feeding behavior after rats are given a free choice between a mixture of chocolates and standard chow food for a limited and unlimited time. This test allows measuring food intake and chocolate preference [38]. The test was performed at adolescence (8–9 postnatal weeks) and it was reevaluated at adulthood (12–13 postnatal weeks) in both perinatal groups and both sexes.

At the beginning of the test, the rats were individually housed. They were then provided with the two types of food and water *ad libitum* during day 1 and 2. In day 3 and 4, they had limited chocolate access (only one hour during the light phase) with standard chow food and water *ad libitum*. At the end of the test, animals were returned to their cages and diets. Caloric intake relative to body weight and chocolate preference (calculated as the percentage of chocolate intake on overall intake) during the test were evaluated in both perinatal groups and sexes and in the two different age periods (adolescence and adulthood).

#### AM251 test: determination of the effect of an acute dose of AM251 on caloric intake of two different types of food in offspring

At adulthood (13–14 postnatal weeks), the differential response to AM251 (an inverse agonist of CB1 receptors) and its effect on caloric intake of two different types of food (standard chow and chocolate mixture) was evaluated. The experiment was performed in both perinatal groups and sexes following a similar procedure to the previously described [43,44]. The day before the test, all animals were food-deprived for 20 hours. At the beginning of the test, the rats received AM251 or vehicle. AM251 (Tocris, Bioscence, Bristol, UK) was dissolved in a vehicle of 5% Tween 80 and 95% of saline and was administered intraperitoneally (i.p.) at a dose of 3 mg/kg. Thirty minutes after administering the compound, animals were placed in an individual cage without bedding material. Then, they were provided with two small cans containing both types of weighed food: standard chow pellets and highly palatable food pellets (composed by the mixture of chocolates described above). Cumulative caloric intake relative to body weight of both types of food and the sum of both (total food intake) were calculated for 240 minutes after the beginning of the test.

### Sample collection

At the 5^th^ postnatal month, animals from both sexes and experimental groups were weighed immediately before death. Then, they were sacrificed by decapitation after administration of Equitesin^®^ (i.p.) (3 mg/kg). This process was carried out in the 2 following hours after the beginning of the dark phase in a separate room from the other experimental animals. Trunk blood samples were briefly collected into tubes containing EDTA (6%) and centrifuged (1500 *g* for 10 minutes at 4°C). The clear layer of plasma was removed, frozen and stored at −80°C for biochemical and hormonal analysis. White perirenal and perigonadal fat were removed and weighed. The weights of the individual fat depots were used to determine the total abdominal fat mass. The whole brain was also removed and immediately frozen in isopentane and stored at −80°C. Then, the hypothalamus was sectioned according to the rat brain atlas of Paxinos and Watson (1998) [45] by a punch done at coronal planes, and immediately processed for mRNA isolation. The hypothalamic area analyzed was ventral to the thalamus, caudal to preoptic area and dorsal to the mammillary bodies. Additionally, samples from perirenal adipose tissue (PAT) and liver were dissected and immediately frozen at −80°C until RT-qPCR analysis.

### Measurement of metabolites, hepatic enzymes and leptin in plasma

The following plasma metabolites were measured: basal glucose, triglycerides, total cholesterol, high-density lipoprotein (HDL), urea, bilirubin, alkaline phosphatase (ALKP), and the hepatic enzymes alanine aminotransferase (ALT), aspartate aminotransferase (AST) and gamma-glutamyltranspeptidase (γGT). These metabolites were analyzed using commercial kits according to the manufacturer’s instructions and a Hitachi 737 Automatic Analyzer (Hitachi Ltd, Tokyo, Japan). Very low-density lipoprotein (VLDL) was estimated following the Friedewald equations [46] and low-density lipoprotein (LDL) was determined by the modification of Friedewal equation proposed by Ahmadi et al. [47]: (VLDL = TG/5); LDL = [(TChol/1.19)+(TG/1.9)- (HDL/1.1)-38]. The leptin levels in plasma were measured using a commercial rat insulin ELISA kit (Cat. no. RD291001200R; BioVendor, Brno, Czech Republic).

### Measurement of body composition

Adiposity was estimated by calculating the percentage of abdominal fat weight over total body weight before sacrifice. The perirenal and perigonadal fat deposits were dissected and weighed as previously described. The sum of both types of fat was used to determine the percentage of abdominal fat.

### RNA isolation and real-time quantitative PCR analysis

We performed real-time qPCR (TaqMan, Life Technologies) as described previously [48]. Tissue portions (100–300 mg) of the liver, PAT and the whole hypothalamus were homogenized. RNA was extracted using the Trizol^®^ method according to the manufacturer’s instruction (Gibco BRL Life Technologies). Reverse transcription was carried out from 1 μg of RNA using the Transcriptor Reverse Transcriptase kit and random hexamer primers (Transcriptor RT, Roche). Real-time qPCR was performed using a CFX96 Touch^™^ Real-Time PCR Detection System (Bio-Rad). The primers used were obtained based on TaqMan^®^ Gene Expression Assays and the FAM^™^ dye label format (Life Technologies). Primers for the PCR reaction (S2 Table) were obtained based on Applied Biosystems’ genome database of rat mRNA references (http://bioinfo.appliedbiosystems.com/genome-database/gene-expression.html). Absolute values from each sample were normalised with regard to the housekeeping gene *Actb*. The relative quantification was calculated using the ΔΔCt method and normalised to the control group.

### Statistical analysis

All data are expressed as mean ± S.E.M. Statistical analysis of results was performed by using the GraphPad Prism version 6.0 program (GraphPad Software Inc., San Diego, CA, USA) and SPSS 20.0 for windows (SPSS Inc., Chicago, IL, USA). Absolute body weight over time and caloric intake were analyzed by three-way repeated measures analysis of variance (ANOVA) with time, perinatal diet and sex as factors. Multiple comparisons were assessed by Bonferroni *post-hoc* test. Results from the compulsive feeding test were analyzed by three-way repeated measures ANOVA with perinatal group (control *vs* free-choice animals), sex and time as factors. Data from the AM251 test were analyzed by two-way ANOVA with treatment and perinatal diet as factors. Further analyses were performed by using one or two-way ANOVA, with perinatal diet and sex as variables, followed by Bonferroni *post-hoc* test for multiple comparisons. A *p*-value below 0.05 was considered statistically significant.

## Results

### Evaluation of the rat dams’ preference for a highly palatable food

**Fig 2:**
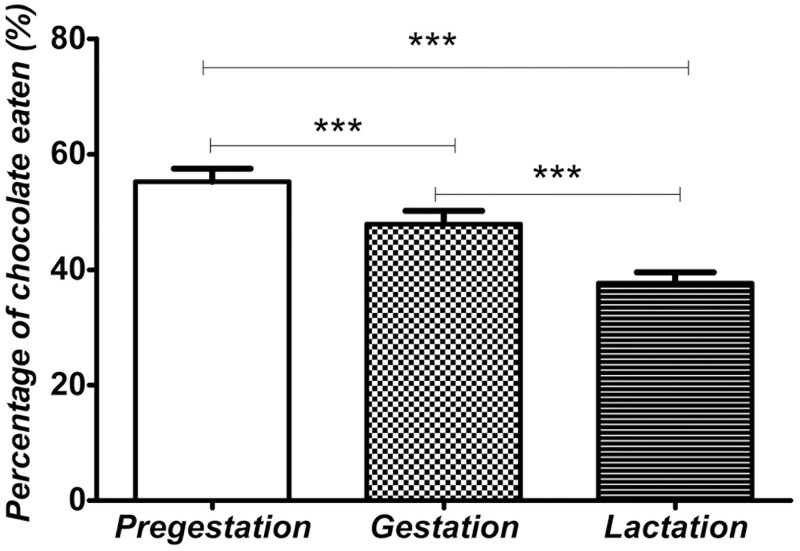
Chocolate preference in rat dams assigned to free choice diet. The bars indicate the average of chocolate preference during every perinatal period (pregestation, gestation and lactation). Values are expressed as means ± S.E.M. Statistical analysis was performed by one-way ANOVA of repeated measures. Bonferroni multiple comparisons indicated significantly differences between groups: *** *p*<0.001.

Rat dams assigned to a free choice diet were evaluated to assess the average of chocolate preference during pregestation, gestation and lactation. The chocolate preference between the three periods was compared. The one-way ANOVA showed that chocolate preference was significantly different between the periods studied (F_*2*,*20*_ = *54*.*699*, *p<0*.*001*) (Fig 2). Specifically, Bonferroni multiple comparisons showed that chocolate preference in the pregestational period was higher than chocolate preference during the gestational period (*t = 4*.*347*, *p<0*.*001*) and lactation period (*t = 10*.*41*, *p<0*.*001*). Additionally, chocolate preference in gestation was higher than chocolate preference in lactation (*t = 10*.*23*, *p<0*.*001*). These data indicate that the preference for a highly palatable food in rat dams assigned to free choice was modified during the perinatal period. Thus, chocolate preference was reduced in gestation. Furthermore, the lowest chocolate preference was reached during the lactation period (Fig 2).

### Evaluation of feeding behavior and food preferences in offspring

#### Compulsive feeding test

**Fig 3:**
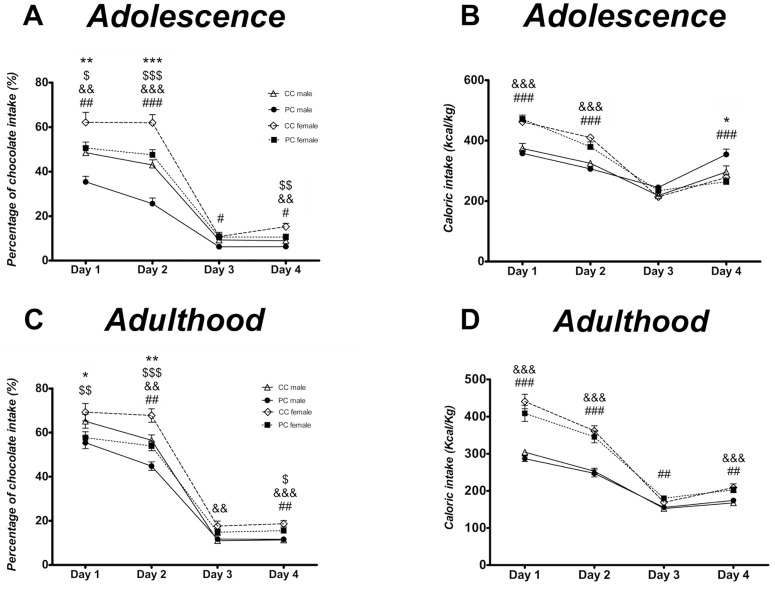
Compulsive feeding test. A,B: chocolate preference and caloric intake during the days of compulsive feeding test at adolescence. C,D: chocolate preference and caloric intake during the days of compulsive feeding test at adulthood. Control male offspring are represented as open triangles, control female as open rhombuses; free choice male offspring represented as solid circles and free choice female offspring as solid squares. Values are expressed as mean ± S.E.M. Three-way repeated measures ANOVA (time, sex and perinatal diet as factors) and Bonferroni *post-hoc* test: */**/***p<0.*05/0*.*01/0*.*001* to compare CC *vs* PC males; ^$/$ $/$ $ $^ *p<0*.*05/0*.*01/0*.*001* to compare CC *vs* PC females; ^&&/&&&^ *p<0*.*01/0*.*001* to compare CC males *vs* females; ^#/##/###^ *p<0*.*05/0*.*01/0*.*001* to compare PC males *vs* females.

Regarding adolescence and chocolate preference (Fig 3A), a three-way repeated measures ANOVA indicated a main effect of time (*F*_*3*,*58*_ = *401*.*884*, *p<0*.*001*), perinatal diet (*F*_*1*,*60*_ = *26*.*463*, *p<0*.*001*) and sex (*F*_*1*,*60*_ = *43*.*511*, *p<0*.*001*). Bonferroni multiple comparisons indicated that the offspring from P dams preferred chocolate less than the control offspring in both sexes (*F*_*1*,*60*_ = *14*.*482*, *p<0*.*001* and *F*_*1*,*60*_ = *11*.*987*, *p<0*.*01*, for male and female offspring respectively). Moreover, female offspring displayed higher chocolate preference than male offspring in both perinatal groups *(F*_*1*,*60*_ = *17*.*002*, *p<0*.*001* and *F*_*1*,*60*_ = *28*.*071*, *p<0*.*001*, for control and free choice perinatal diet respectively). Interaction between time, perinatal diet and sex were not significant. However, the Bonferroni test indicated that differences between male CC and male PC were significant the days with free access to chocolate (days 1 and 2) whereas the differences between female CC and female PC were significant on day 4 as well (when rats had limited access to highly palatable food). Additionally, female offspring showed higher preference for chocolate than male offspring every day of the test in the free choice group, and 3 days of the test in the control group (Fig 3A).

Regarding to caloric intake during adolescence (Fig 3B), a three-way ANOVA showed a main effect of time (*F*_*3*,*58*_ = *267*.*177*, *p<0*.*001*), sex *(F*_*1*,*60*_ = *10*.*605*, *p<0*.*01)*, interaction between time and perinatal diet (*F*_*3*,*58*_ = *3*.*913*, *p<0*.*05)* and interaction between time and sex (*F*_*3*,*58*_ = *27*.*710*, *p<0*.*001*). No significant effect of perinatal diet was found. Specifically, Bonferroni multiple comparisons indicated that female offspring from the C group presented higher caloric intake relative to body weight than male offspring from C dams (*F*_*1*,*60*_ = *7*.*691*, *p<0*.*01*). A similar tendency was noticed in P perinatal group (*F*_*1*,*60*_ = *3*.*163*, *p = 0*.*08)*. It was found that the differences between the male and female offspring of control dams were significant on the first 2 days with free access to chocolate (^&&&^ *p<0*.*001*). In contrast, the differences between male and female PC were significant on days 1, 2 and 4 (^*###*^ *p<0*.*001*). Male offspring from P group displayed higher caloric intake than males from C group at day 4 of the test (* p<0.*05*) (Fig 3B).

Concerning chocolate preference in adulthood (Fig 3C), we detected an effect of time (*F*_*3*,*61*_ = *587*.*130*, *p<0*.*001*), perinatal diet (*F*_*1*,*63*_ = *18*.*156*, *p<0*.*001* and sex (*F*_*1*,*63*_ = *15*.*384*, *p<0*.*001*). The interaction between time and perinatal diet, and the interaction between time and sex were also significant (*F*_*3*,*61*_ = *10*.*031*, *p<0*.*001* and *F*_*3*,*61*_ = *3*.*372*, *p<0*.*05*, respectively). Similar to the adolescent period, offspring from P perinatal group displayed lower chocolate preference than control offspring in both sexes (*F*_*1*,*63*_ = *5*.*296*, *p<0*.*05* and *F*_*1*,*63*_ = *14*.*311*, *p<0*.*001*, for male and female respectively). Furthermore, the increased preference for chocolate in female offspring was also demonstrated in adulthood in both perinatal groups (*F*_*1*,*63*_ = *10*.*492*, *p<0*.*01* and *F*_*1*,*63*_ = *5*.*129*, *p<0*.*05*, for control and free choice perinatal groups respectively). Additionally, differences between CC and PC groups were significant in male offspring on the days when chocolate was fully available (*/** *p<0*.*05/0*.*01*, for days 1 and 2 respectively). In contrast the difference between female CC and female PC was significant on days 1, 2 and 4 (^*$/$ $/$ $ $*^ *p<0*.*05/0*.*01/0*.*001)*. Regarding sex differences, the Bonferroni test indicated significant differences from day 2 to 4 in the control perinatal group (^&&/&&&^ *p<0*.*01/0*.*001*). In contrast, significant differences were found between male and female from P dams on days 2 and 4 (^##^ *p<0*.*01*) (Fig 3C).

In relation to the caloric intake during adulthood, a three-way repeated measures ANOVA indicated a main effect of time (*F*_*3*,*61*_ = *176*.*469*, *p<0*.*001)*, sex (*F*_*1*,*63*_ = *113*.*131*, *p<0*.*001*) and a significant interaction between time and sex (*F*_*3*,*61*_ = *14*.*581*, *p<0*.*001*). Specifically, female offspring presented higher caloric intake relative to body weight than male offspring in both perinatal groups (*F*_*1*,*63*_ = *56*.*683*, *p<0*.*001* and *F*_*1*,*63*_ = *56*.*756*, *p<0*.*001* for control and free choice perinatal groups respectively) (Fig 3D).

Taken together, these data indicate that in both periods of time (adolescence and adulthood) P offspring from both sexes displayed lower chocolate preference than control offspring during different days of the test. Additionally, females from both perinatal groups exhibited greater chocolate preference. Concerning caloric intake, female offspring presented higher caloric intake relative to body weight as well in both periods of time. However, there were no significant differences between the caloric intake of the perinatal groups throughout the test in both periods of time studied.

#### AM251 test

**Fig 4:**
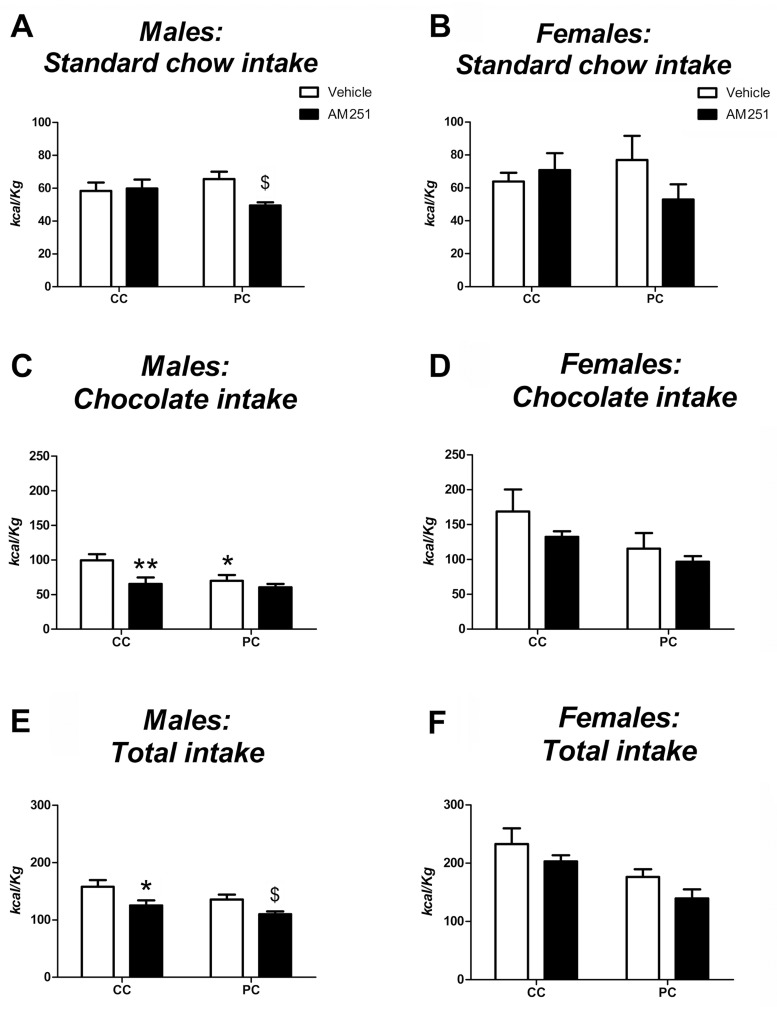
AM251 test performed at adulthood. A-F: Standard chow intake, chocolate intake and total intake (the sum of chocolate and standard chow intake) relative to body weight after 240 minutes of the beginning of the AM251 test in male (**A,C,E**) and female offspring (**B,D,F**). Values are expressed as mean ± S.E.M. Open bars indicate vehicle treatment. Solid bars denote AM251 treatment. Two-way ANOVA and Bonferroni *post-hoc* test: */** *p<0*.*05/0*.*01 vs* CC vehicle group; ^$^ *p<0*.*05 vs* PC vehicle group.

Regarding male offspring and standard chow intake (Fig 4A), a two-way ANOVA revealed no significant effect of both treatment and perinatal diet. However, the interaction between perinatal diet and treatment was almost significant (*F*_*1*,*26*_ = *4*.*006*, *p = 0*.*056*). Specifically, Bonferroni multiple comparisons showed that male offspring from the free choice perinatal group treated with AM251 displayed lower standard food intake than control offspring (*F*_*1*,*26*_ = *6*.*698*, *p<0*.*05*) (Fig 4A). Regarding chocolate intake (Fig 4C), a two-way ANOVA showed a significant main effect of perinatal diet and treatment. Particularly, control offspring exhibited greater cumulative chocolate intake than free choice offspring (*F*_*1*,*26*_ = *4*.*835*, *p<0*.*05*). Additionally, animals who received AM251 displayed decreased chocolate intake than vehicle animals (*F*_*1*,*26*_ = *7*.*685*, *p<0*.*05*). Although the interaction between perinatal diet and treatment was not significant, Bonferrroni multiple comparisons revealed that male offspring from the control perinatal group treated with AM251 displayed lower intake than vehicle animals from the same perinatal group (*F*_*1*,*26*_ = *9*.*466*, *p<0*.*01*), whereas no significant differences were found in free choice offspring. Additionally, free choice offspring who were administered the vehicle exhibited lower chocolate intake than control offspring with the same treatment (*F*_*1*,*26*_ = *6*.*689*, *p<0*.*16*) (Fig 4C). Analyzing the variable total intake (Fig 4E), the two-way ANOVA indicated a main effect of treatment (*F*_*1*,*26*_ = *11*.*442*, *p<0*.*01*) and perinatal diet (*F*_*1*,*26*_ = *4*.*761*, *p<0*.*05*). Thus, PC animals exhibited lower intake than CC males. Additionally, Bonferroni multiple comparisons showed that animals injected with AM251 displayed lower total intake in both perinatal groups (*F*_*1*,*26*_ = *7*.*205*, *p<0*.*05* and *F*_*1*,*26*_ = *4*.*411*, *p<0*.*05* for control and free choice perinatal groups respectively) (Fig 4E).

Concerning female offspring and standard chow intake (Fig 4B), a two-way ANOVA did not show significant effects of either on treatment or perinatal diet. However, Bonferroni multiple comparisons indicated a tendency to a decreased standard chow intake in PC females treated with AM251 compared to vehicle ones (*F*_*1*,*24*_ = *3*.*447*, *p = 0*.*076*) (Fig 4B). Regarding chocolate intake (Fig 4D), a tendency effect of perinatal diet (*F*_*1*,*24*_ = *4*.*291*, *p<0*.*058*) was observed. Thus, control female offspring exhibited higher chocolate intake than P female offspring. However, no significant effect of treatment was found (Fig 4D). Regarding the total intake (Fig 4F), ANOVA showed a main effect of treatment (*F*_*1*,*24*_ = *6*.*382*, *p<0*.*05*) and perinatal diet (*F*_*1*,*24*_ = *7*.*407*, *p<0*.*05*). Indeed, females injected with AM251 displayed a decreased total intake compared to vehicle females (*F*_*1*,*24*_ = *6*.*382*, *p<0*.*05*), and PC females exhibited lower intake than CC females (*F*_*1*,*24*_ = *7*.*407*, *p<0*.*05*). Additionally, the Bonferroni test indicated a higher tendency for PC females treated with AM251 to decrease total intake compared to vehicle ones (*F*_*1*,*24*_ = *4*.*276*, *p = 0*.*05*) (Fig 4F).

These results show that offspring treated with AM251 displayed a lower total food intake relative to body weight in both sexes. However, there were differences in the type of food consumed depending on the perinatal diet and sex. Thus, PC male offspring treated with AM251 displayed lower total food intake and standard food intake than vehicle ones, whereas CC males treated with AM251 presented lower total food intake and chocolate intake than vehicle ones. Regarding female offspring, PC females given AM251 showed a tendency to decrease total and standard food intake, similar to PC males. However, no differences were found when compared to CC females in the studied variables.

### Effect of maternal diet on body weight and caloric intake in offspring until adulthood

**Fig 5:**
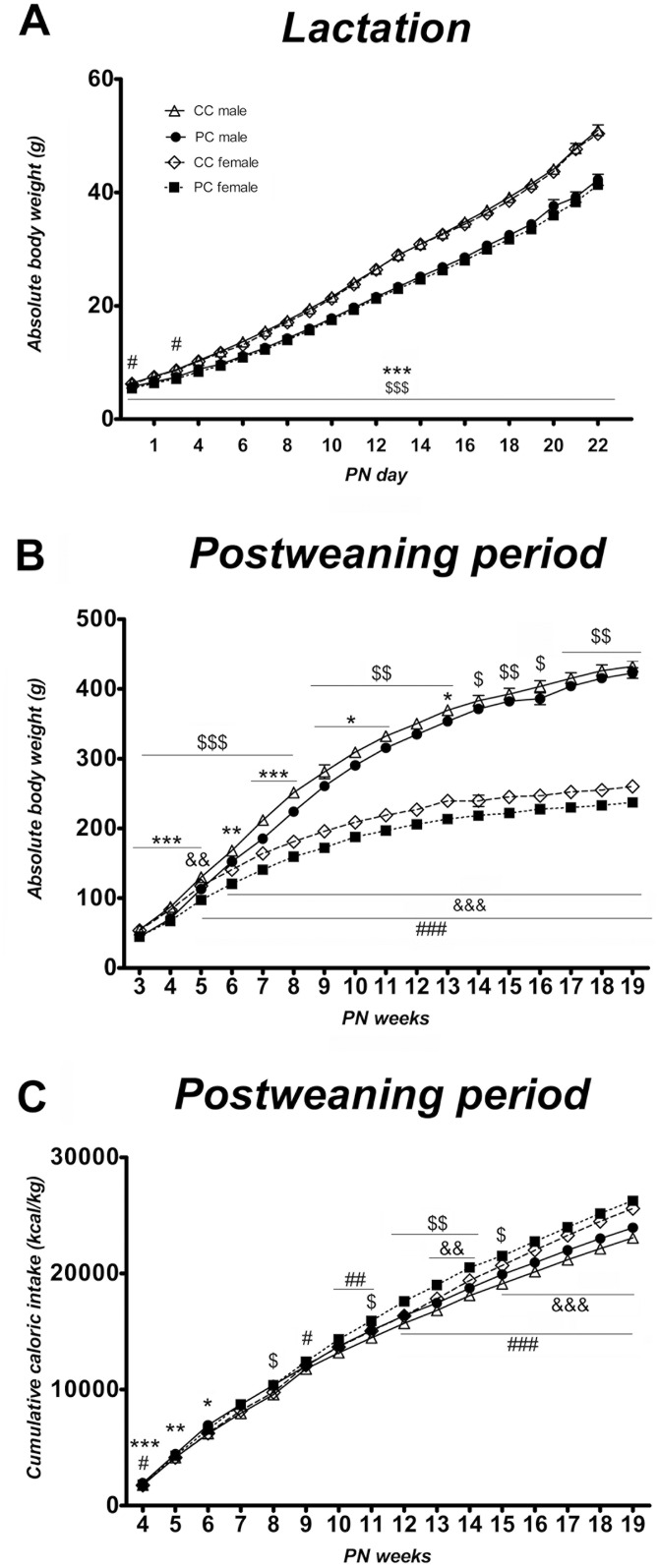
Effect of perinatal diet on body weight and caloric intake of offspring. Absolute body weight (g) of control offspring and free choice offspring from both sexes during lactation **(A)** and postweaning period **(B)**. Cumulative caloric intake (kcal/kg) of control offspring and free choice offspring from both sexes during postweaning period **(C)**. Control male offspring are represented as open triangles, control female as open rhombuses, free choice male offspring are represented as solid circles and free choice female offspring as solid squares. Values are expressed as mean ± S.E.M. Three-way repeated measures ANOVA (time, sex and perinatal diet as factors) and Bonferroni *post-hoc* test: */**/*** *p<0*.*05/0*.*01/0*.*001* to compare CC *vs* PC males; ^$/$ $/$ $ $^ *p<0*.*05/0*.*01/0*.*001* to compare CC *vs* PC females; ^&&/&&&^ *p<0*.*05/0*.*01/0*.*001* to compare CC males *vs* females; ^#/##/###^ *p<0*.*05/0*.*01/0*.*001* to compare PC males vs females.

Offspring body weight evolution was assessed during the lactation and postweaning periods. From birth to PN day 22, a three-way repeated measures ANOVA showed an effect of time (*F*_*22*,*129*_ = *738*.*268*, *p<0*.*001*), perinatal diet (*F(*_*1*,*150*_*) = 11750*.*56*, *p<0*.*001*), and interaction between time and perinatal diet (*F*_*22*,*129*_ = *11*.*985*, *p<0*.*001*). Male and female offspring from free choice dams displayed lower weight during the entire lactation period (*F*_*1*,*150*_ = *51*.*790*, *p<0*.*001* and *F*_*1*,*150*_ = *61*.*532*, *p<0*.*001*, respectively). Indeed, Bonferroni multiple comparisons showed significant differences between perinatal groups in both sexes from birth to PN day 22 (Fig 5A). Regarding the post-weaning period, a three-way repeated measures ANOVA showed an effect of time (*F*_*16*,*50*_ = *888*.*939*, *p<0*.*001*, perinatal diet (*F*_*1*,*65*_ = *18*.*290*, *p<0*.*001*) and sex (*F*_*1*,*65*_ = *538*.*791*, *p<0*.*001*). The interaction between time, sex and perinatal diet was also significant (*F*_*16*,*50*_ = *3*.*101*, *p<0*.*01*). Specifically, the Bonferroni test showed that male offspring from P dams was leaner than control male offspring until the end of adolescence/beginning of adulthood (11-13^th^ PN weeks). In contrast, female offspring from P dams presented lower weight during all stages. Additionally, sexual differences in body weight started to be significant at the 5^th^ postnatal week in both perinatal groups (Fig 5B).

Concerning caloric intake relative to body weight, we found a significant effect of time (*F*_*15*,*975*_ = *15*,*11731*, *p<0*.*001*), perinatal diet (*F*_*1*,*65*_ = *4*.*821*, *p<0*.*05*), sex (*F*_*1*,*65*_ = *32*.*42*,*3 p<0*.*001*) and a significant interaction between time, perinatal diet and sex (*F*_*15*,*975*_ = *2*.*321*, *p<0*.*01*). Bonferroni multiple comparisons indicated that male offspring from free choice dams exhibited hyperphagia during the first weeks of infant period when they were compared to control male offspring (*/**/*** p<0.*05/0*.*01/0*.*001*). Moreover, female offspring from P dams displayed hyperphagia at the 8^th^ week and from the 11 to 15^th^ week of adolescence (^*$/$ $*^ *p<0*.*05/0*.*01*). Additionally, female offspring displayed higher caloric intake relative to body weight than male offspring in both perinatal groups (Fig 5C).

Together these results indicate that offspring from free choice perinatal group exhibited lower weight and hyperphagia than their controls in different stages of development. Furthermore, data denotes that these effects depend on the sex. Thus, male offspring displayed these findings during infant period and adolescence whereas female offspring kept a lower weight during all stages and showed hyperphagia in adolescence up to the 15^th^ PN week.

### Effect of maternal diet on leptinemia, plasmatic metabolites and adiposity of offspring at adulthood

A two-way ANOVA showed an effect of sex on the plasma levels of leptin, glucose, HDL, AST and AST/ALT ratio (Table 1). We also detected an effect of perinatal diet on the plasma levels of triglycerides, HDL, LDL, VLDL and bilirubin. An interaction between factors was only found in the plasma levels of leptin. Bonferroni multiple comparisons demonstrated that PC males exhibited higher levels of leptin, LDL and bilirubin, but lower levels of HDL, than CC males (*/** p<0.*05/0*.*01*). Equally, we observed higher levels of LDL, but lower levels of HDL in PC females compared to CC females (^*$/$ $*^ *p<0*.*05/0*.*01*). Additionally, PC females displayed lower levels of leptin, glucose, total cholesterol, and AST/ALT ratio than PC males *(*^*#/###*^ *p<0*.*05/0*.*001*) (Table 1).

**Table 1:** Plasma leptin, metabolites and hepatic transaminases at 5^th^ postnatal month^a^.

	Male		Female		Two-way ANOVA		
	CC (n = 6)	PC (n = 5)	CC (n = 5)	PC (n = 5)	Interaction	Perinatal diet	Sex
**Leptin (μg/mL)**	5.829±0.88	11.21 ±1.69*	6.07±2.17	**4.15±0.89**^#^	*F*_1,16_ = 6.817, *p* = 0.0189	NS	*F*_1,16_ = 5.94, *p* = 0.0269
**Glucose (mg/dL)**	187.33±12.11	211.40±17.04	169.60±15.65	**154.80±15.57**^#^	NS	NS	*F*_1,17_ = 7.60, *p* = 0.013
**Triglycerides (mg/dL)**	79.50±3.12	105.40±9.84	82.60±15.96	110.40±10.87	NS	*F*_1,17_ = 8.241, *p* = 0.011	NS
**tCholesterol (mg/dL)**	39.00±5.49	38.60±2.61	37.20±2.53	**26.40±4.04**^#^	NS	NS	NS
**HDL (mg/dL)**	26.67±3.04	13.80±7.73*	21.60±2.91	**2.20±0.82**^$ $^	NS	*F*_1,17_ = 17.304, *p* = 0.001	F_1,17_ = 4.617, *p* = 0.046
**LDL (mg/dL)**	12.37±2.79	37.36±11.80*	17.01±8.4	**40.29±4.76**^$^	NS	*F*_1,17_ = 13.144, *p* = 0.002	NS
**VLDL (mg/dL)**	15.90±0.62	21.08±1.97	16.52±3.19	22.08±2.17	NS	*F*_1,17_ = 8.241, *p* = 0.011	NS
**Urea (mg/dL)**	27.50±2.89	26.80±1.29	30.40±2.02	28.80±4.33	NS	NS	NS
**Bilirubin (mg/dL)**	0.13±0.02	0.50±0.19**	0.20±0.05	0.24±0.06	NS	F_1,17_ = 5.512, *p* = 0.031	NS
**ALKP (UI)**	73.83±4.57	64.60±2.66	63.20±7.79	68.40±2.08	NS	NS	NS
**γGT (UI)**	10.50±1.01	8.80±1.78	12.20±0.96	11.40±0.57	NS	NS	NS
**AST (UI)**	162.50±33.02	108.80±18.26	145.00±20.39	172.60±27.22	NS	NS	F_1,17_ = 5.152, *p* = 0.037
**ALT (UI)**	55.00±3.96	59.80±6.26	70.40±8.37	74.60±10.56	NS	NS	NS
**AST/ALT**	0.39±0.06	0.57±0.38	4.05±0.24***	**3.97±0.42**^###^	NS	NS	*F*_1,17_ = 290.791, *p*<0.001

^a^Values are expressed as means ± S.E.M. Statistical analysis was performed by two-way ANOVA with perinatal diet and sex as factors.

CC: offspring from control-fed dams. PC: offspring from highly palatable diet-fed dams. NS: non significant.

Bonferroni test: */**/***p <0.05/0.01/0.001 *vs* CC males;

^$/$ $^*p*<0.05/0.01 *vs* CC females;

^#/###^*p*<0.05/0.001 *vs* PC males.

Concerning adiposity (Table 2), we found an effect of sex on perirenal, perigonadal and abdominal fat, and perirenal and perigonadal fat percentage relative to body weight. An effect of perinatal diet on perirenal, perigonadal and abdominal fat, as well as their respective percentage relative to body weight was found. Interaction between sex and perinatal diet was specifically observed in perirenal fat weight and the percentage of perirenal fat relative to body weight. Particularly, PC males exhibited an increase in perirenal and abdominal fat weights as well as their respective percentages relative to body weight compared to CC males (**/*** p<0.*01/0*.*001*). PC females only showed an increase in perigonadal fat percentage with respect to CC females (^*$*^ *p<0*.*05*). Moreover, CC and PC females exhibited a decrease in the perirenal, perigonadal and abdominal fat weights compared to the respective CC (**/*** p<0.*01/0*.*001*) and PC (^*##/###*^ *p<0*.*01/0*.*001*) males (Table 2).

**Table 2:** Adiposity at 5^th^ postnatal month^a^.

	Male		Female		Two-way ANOVA		
	CC (n = 12)	PC (n = 10)	CC (n = 11)	PC (n = 11)	Interaction	Perinatal diet	Sex
**Perirenal fat (g)**	8.90±0.41	12.04±0.65***	4.82±0.36***	**4.92±0.43**^###^	*F*_1.40_ = 10.661 *p* = 0.002	*F*_1,40_ = 12.309 *p* = 0.001	*F*_1.40_ = 144.049 *p*<0.001
**Perirenal fat/BW (%)**	1.97±0.08	2.42±0.26***	1.80±0.12	**1.94±0.15**^###^	*F*_1,40_ = 5,170, *p* = 0.028	*F*_1,40_ = 11.497 *p* = 0.002	*F*_1,40_ = 13.570, *p* = 0.001
**Perigonadal fat (g)**	9.32±0.61	11.20±1.15	6.14±0.67**	**7.53±0.51**^##^	NS	*F*_1,40_ = 4.730, *p* = 0.036	*F*_1,40_ = 20.894, *p*<0.001
**Perigonadal fat/BW (%)**	2.06±0.12	2.25±0.30	2.29±0.21	**2.97±0.16**^$^	NS	*F*_1,40_ = 8.466, *p* = 0.006	*F*_1,40_ = 4.025, *p* = 0.052
**Abdominal fat (g)**	18.22±0.94	23.23±1.69**	10.96±0.96***	**12.44±0.87**^###^	NS	*F*_1,40_ = 8.318, *p* = 0.006	*F*_1,40_ = 64.228, *p*<0.001
**Abdominal fat/BW (%)**	4.03±0.18	5.11±0.33**	4.15±0.28	4.90±0.29	NS	*F*_1,40_ = 11.599 *p* = 0.002	NS

^a^Values are expressed as means ± S.E.M. Statistical analysis was performed by two-way ANOVA with perinatal diet and sex as factors.

CC: offspring from control-fed dams. PC: offspring from highly palatable diet-fed dams. NS: non significant.

Bonferroni test: **/***p<0.01/0.001 *vs* CC males;

^$^*p*<0.05 *vs* CC females;

^##/###^*p*<0.01/0.001 *vs* PC males.

In summary, these data indicate that animals perinatally exposed to a highly palatable diet presented alterations in plasmatic metabolites and adiposity. Particularly, PC male offspring displayed higher leptinemia, decreased levels of plasma HDL, increased levels of plasma LDL, greater bilirubin, increased perirenal fat and abdominal adiposity than CC male offspring. In contrast, PC female offspring displayed decreased levels of plasma HDL and increased levels of plasma LDL and perigonadal adiposity when they were compared to CC females. In addition, sexual dimorphism was found in the leptinemia, glycemia, HDL, the AST/ALT ratio and adiposity.

### Effect of maternal diet on endocannabinoid metabolism gene expression on the hypothalamus, liver and PAT of offspring at adulthood

Gene expression levels of several ECS components (*Cnr1*, *Cnr2*, *Napepld*, *Faah*, *Daglα*, *Daglβ*, *Mgll*) were evaluated in male and female offspring from rat dams fed either a standard chow (CC) or a high caloric diet (PC) in the hypothalamus, liver and perirenal adipose tissue (PAT). We decided to evaluate the endocannabinoid metabolism gene expression in PAT based on the prominent adiposity observed in PC male offspring.

**Fig 6:**
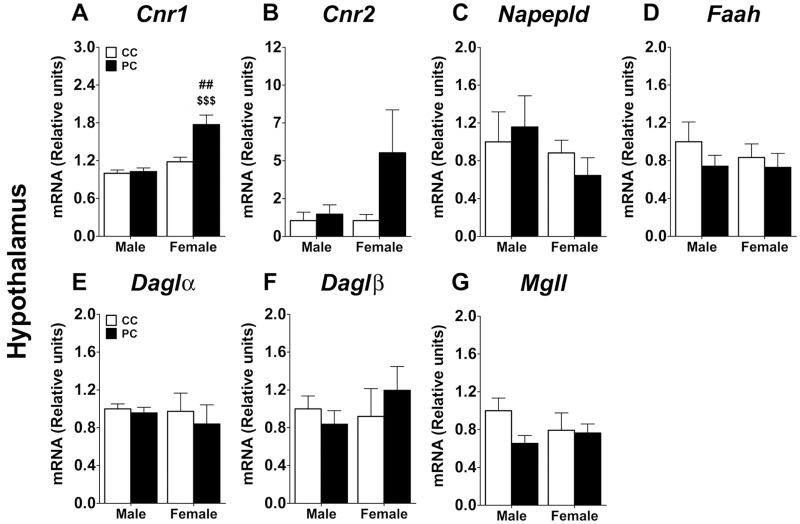
Levels of endocannabinoid metabolism gene expression in the hypothalamus. **A**.-*Cnr1*, cannabinoid CB1 receptor; **B**.-*Cnr2*, cannabinoid CB2 receptor; **C**.-*Napepld*, N-acyl phosphatidylethanolamide-dependente phospholipase D; **D**.-*Faah*, fatty acid amidohydrolase; **E**.-*Daglα*, diacyl-glycerol lipase alpha; **F**.- *Daglβ*, diacyl-glycerol lipase beta; G.-*Mgll*, monoacyl-glycerol lipase. Values are expressed as means ± S.E.M. Two-way ANOVA and Bonferroni *post-hoc* test: ^##^ *p<0*.*01 vs* PC male group; ^$ $ $^ *p<0*.*001 vs* CC female group.

Regarding hypothalamus, a two-way ANOVA indicated an effect of perinatal diet *(F*_*1*,*17*_ = *27*.*32*, *p<0*.*0001)* and sex *(F*_*1*,*17*_ = *10*.*01*, *p<0*.*01)*, and an interaction between factors *(F*_*1*,*17*_ = *12*.*09*, *p<0*.*01)* in the gene expression of *Cnr1* (Fig 6A). No effect or interaction was observed on the gene expression of the remaining ECS components (Fig 6B–6G). Regarding the Bonferroni analysis, PC female offspring showed an increase in the gene expression of *Cnr1* in the hypothalamus when they were compared to PC males and CC females respectively *(*^*##/$ $ $*^ *p<0*.*01/0*.*001)*.

**Fig 7:**
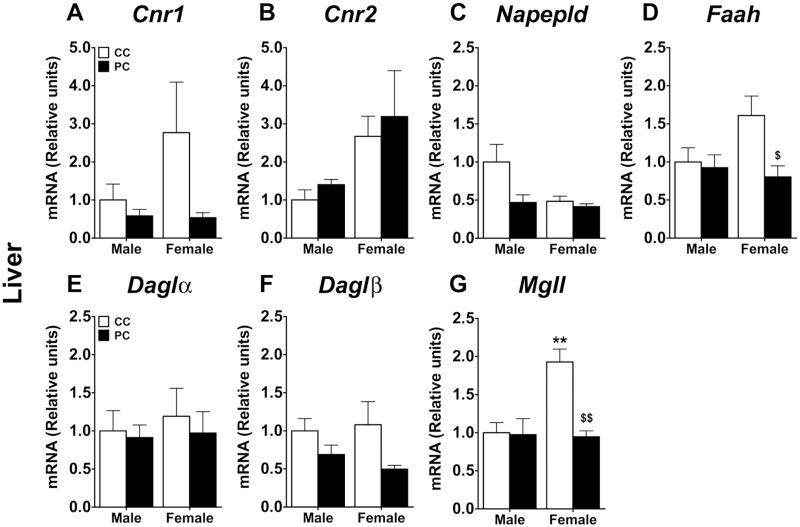
Levels of endocannabinoid metabolism gene expression in liver. Values are expressed as means ± S.E.M. Two-way ANOVA and Bonferroni *post-hoc* test: ** *p<0*.*01 vs* CC male group; ^$/$ $^ *p<0*.*05/0*.*01 vs* CC female group. For abbreviations see legend to Fig 6 or S2 Table.

Concerning the liver, a two-way ANOVA did not show any effect or interaction on the gene expression of *Cnr1* and *Daglα* (Fig 7A and 7E). However, we detected a sex effect on the gene expression of *Cnr2 (F*_*1*,*17*_ = *7*.*15*, *p = 0*.*016)*, *Napepld (F*_*1*,*16*_ = *4*.*65*, *p = 0*.*0465)* and *Mgll (F*_*1*,*17*_ = *8*.*55*, *p = 0*.*009)* (Fig 7B, 7C and 7G). A perinatal diet effect on the expression of *Napepld (F*_*1*,*16*_ = *5*.*16*, *p = 0*.*0372)*, *Faah (F*_*1*,*17*_ = *5*.*19*, *p = 0*.*036)*, *Daglβ (F*_*1*,*16*_ = *5*.*93*, *p<0*.*05)* and *Mgll (F*_*1*,*17*_ = *10*.*73*, *p<0*.*001)* was also found (Fig 7C, 7D, 7F and 7G). Interaction between factors was only detected in the gene expression of *Mgll (F*_*1*,*17*_ = *9*.*62*, *p = 0*.*0065)* (Fig 7G). Specifically, Bonferroni multiple comparisons showed that PC female offspring exhibited a decrease in the mRNA levels of *Faah* and *Mgll* when they were compared to CC females *(*^*$/$ $*^ *p<0*.*05/0*.*01*) (Fig 7D and 7G). *Mgll* gene expression was increased in CC female offspring when they were compared to CC males (** p<0.*01*) (Fig 7G).

**Fig 8:**
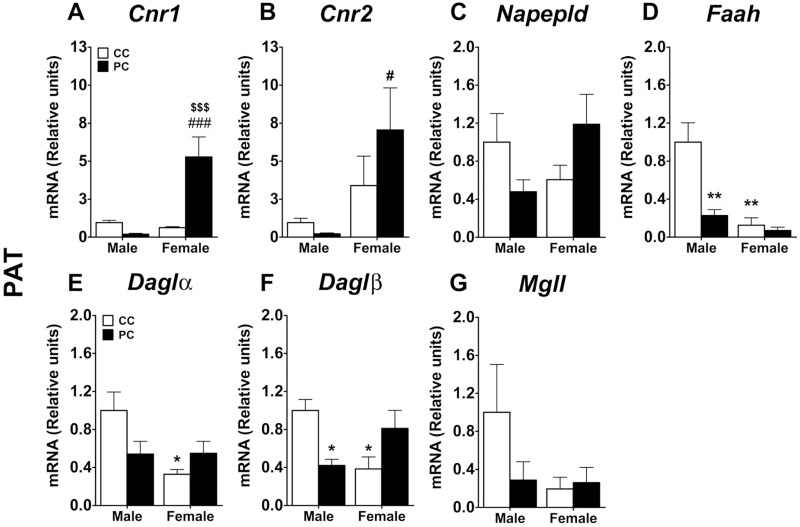
Levels of endocannabinoid metabolism gene expression in perirenal adipose tissue (PAT). Values are expressed as means ± S.E.M. Two-way ANOVA and Bonferroni *post-hoc* test: */** *p<0*.*05/0*.*01 vs* CC male group; ^#/###^ *p<0*.*05/0*.*001 vs* PC male group; ^$ $ $^ *p<0*.*001 vs* CC female group. For abbreviations see legend to Fig 6 or S2 Table.

In relation to PAT, a two-way ANOVA indicated an effect of perinatal diet *(F*_*1*,*16*_ = *13*.*8*, *p = 0*.*0018*) and sex *(F*_*1*,*16*_ = *20*.*73*, *p = 0*.*003*), and an interaction between factors *(F*_*1*,*16*_ = *27*.*08*, *p<0*.*0001)* on the gene expression of *Cnr1*. Particularly, a Bonferroni analysis indicated that PC female offspring showed an increase in the gene expression of *Cnr1* when they were compare to CC females and PC males *(*^*$ $ $/###*^ *p<0*.*001*) (Fig 8A). A sex effect was also detected on the gene expression of *Cnr2* (*F*_*1*,*16*_ = *9*.*99*, *p = 0*.*0061)*. The Bonferroni test indicated that PC females had an increased gene expression of *Cnr2* when they were compared to PC males (^*#*^ *p<0*.*05*) (Fig 8B). Regarding *Faah*, we detected an effect of perinatal diet *(F*_*1*,*16*_ = *9*.*23*, *p = 0*.*0078)* and sex *(F*_*1*,*16*_ = *14*.*21*, *p = 0*.*0017)*, and an interaction between factors *(F*_*1*,*16*_ = *6*.*89*, *p = 0*.*0184)*. PC male and CC female offspring showed decreased gene expression of *Faah* compared to CC males (** p<0.*01*) (Fig 8D). Concerning *Daglα*, a two-way ANOVA indicated a main effect of perinatal diet (*F*_*1*,*16*_ = *4*.*95*, *p = 0*.*040)* and a significant interaction between factors *(F*_*1*,*16*_ = *5*.*195*, *p = 0*.*037*). A *post-hoc* test indicated that CC females had a decreased gene expression of *Daglα* compared to CC males (Fig 8E). Similarly, an interaction between sex and perinatal diet was found in *Daglβ* gene expression *(F*_*1*,*15*_ = *15*.*84*, *p = 0*.*0012)*. Thus, PC male offspring and CC female offspring presented a decreased gene expression of *Daglβ* when they were compared to CC male offspring (* p<0.*05*) (Fig 8F). No effects and interaction were found in the gene expression of *Napepld* and *Mgll* (Fig 8C and 8G).

Taken together, these results indicate that animals exposed to a highly palatable food during the perinatal period presented modifications in some components of the ECS gene expression later in life, even though they were maintained with a standard chow diet after weaning. These alterations include modifications in the hypothalamus, liver and PAT. Specifically, it has been shown that P offspring exhibited modifications in *Cnr1* gene expression of hypothalamus and PAT, alterations in the expression of *Napepld*, *Daglβ* and *Mgll* in the liver and decreased levels of *Faah* in the liver and PAT. However, sexual differences were found in these findings. Thus, PC females showed an increased expression of *Cnr1* gene expression in the hypothalamus and PAT and reduced expression of *Mgll* in the liver when they were compared to CC females and/or PC males. In contrast, PC males exhibited significantly reduced levels in the gene expression of *Faah* and *Daglβ* in PAT when they were compared to CC males. Additionally, female offspring had an increased expression of *Cnr2* in the liver and PAT, that was significantly different from the *Cnr2* expression of PC males in PAT.

### Effect of maternal diet on fatty acid metabolism and *Ppars* gene expression on the liver and PAT of offspring at adulthood

**Fig 9:**
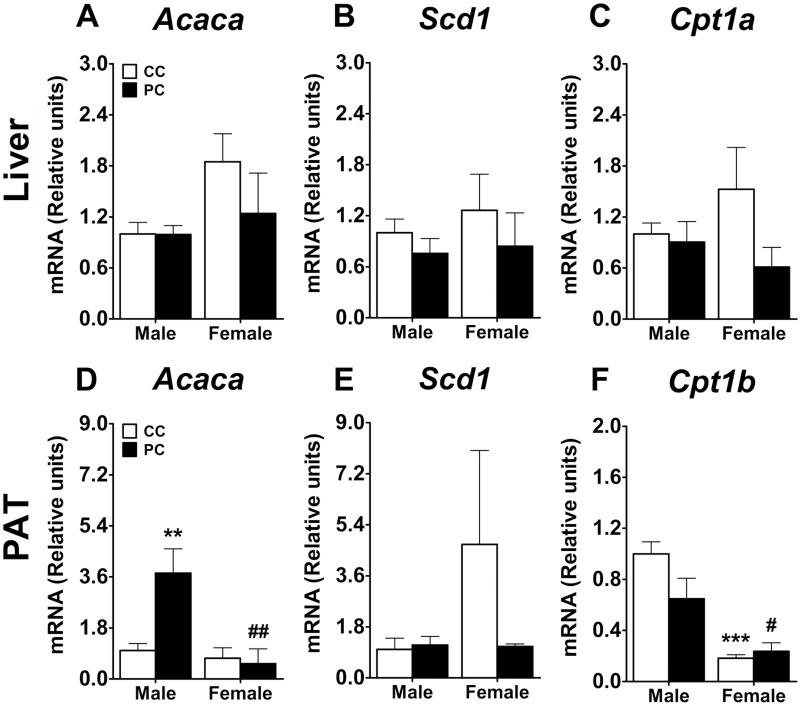
Levels of fatty acid metabolism gene expression in liver (A-C) and PAT (D-F). *Acaca*, acetilcoenzyme A carboxilase; *Scd1*, stearoylconzyme A desaturase 1; *Cpt1a/b*, carnitine palmitoyltrasnferase 1 liver/muscle isoforms. Values are expressed as means ± S.E.M. Two-way ANOVA and Bonferroni *post-hoc* test: **/*** *p*<0.01/0.001 *vs* CC male group; ^#/##^ *p*<0.05/0.01 *vs* PC male group.

The gene expression of selected key metabolic enzymes regulating lipogenesis (*Acaca/Scd1*) and β-oxidation (*Cpt1a*, *Cpt1b*) in the liver and PAT was evaluated in the offspring of rats fed with either a high caloric or a standard chow diet (Fig 9). Regarding the liver, we only detected a main effect of sex on the gene expression of *Cpt1a* (*F*_*1*,*17*_ = *35*.*48*, *p<0*.*0001*) (Fig 9C). In PAT, we observed an effect of perinatal diet (*F*_*1*,*16*_ = *5*.*536*, *p = 0*.*03)* and sex *(F*_*1*_,_*16*_ = *10*.*3*, *p<0*.*01)*, and a significant interaction between factors (*F*_*1*,*16*_ = *7*.*341*, *p = 0*.*0155)* in the gene expression of *Acaca*. Regarding the Bonferroni analysis, PC male offspring showed an increase in the gene expression of *Acaca* in PAT when they were compared to CC males and PC females (**/^*##*^ *p<0*.*01*) (Fig 9D). A two-way ANOVA detected a sex effect on the gene expression of *Cpt1b* in PAT (*F*_*1*,*16*_ = *35*.*48*, *p<0*.*0001)*. Thus, females from both perinatal groups (CC and PC) presented a decrease in the gene expression of *Cpt1b* compared to the respective CC and PC males (^#^/*** *p*<0.05/0.001) (Fig 9F). Interestingly, no effects on *Scd1* gene expression were found in the liver and PAT (Fig 9B and 9E).

**Fig 10:**
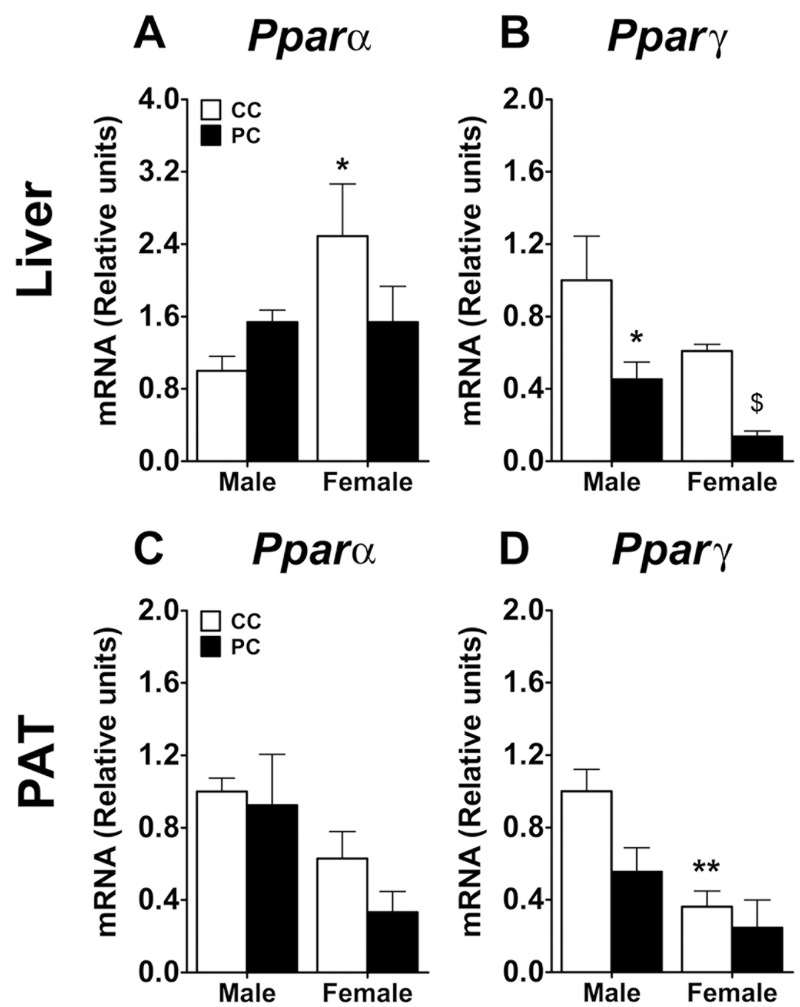
Levels of *Pparα* and *Pparγ* gene expression in liver (A,B) and PAT (C,D). Values are expressed as means ± SEM. Two-way ANOVA and Bonferroni *post-hoc* test: */** *p<0*.*05/0*.*01 vs* CC male group.

Additionally, the gene expression of *Pparα* and *Pparγ* was also analyzed in the liver and PAT (Fig 10). We found a sex effect (*F*_*1*,*17*_ = *4*.*51*, *p = 0*.*0487)* and a significant interaction between factors *(F*_*1*,*17*_ = *4*.*53*, *p = 0*.*0048)* in the gene expression of *Pparα* in the liver. Specifically, the Bonferroni analysis indicated that CC females presented an increased gene expression of *Pparα* when they were compared to CC males (* p<0.*05)* (Fig 10A). Concerning *Pparγ* in the liver, a two-way ANOVA detected a perinatal diet effect (*F*_*1*,*16*_ = *14*.*58*, *p = 0*.*0015)* and a sex effect *(F*_*1*,*17*_ = *4*.*51*, *p = 0*.*048*). Thus, PC male and PC female offspring exhibited a reduced gene expression of *Pparγ* compared to CC males and PC females respectively (*/$ p<0.*05/0*.*05)* (Fig 10B). Regarding PAT, we detected a sex effect on the gene expression of *Pparα (F*_*1*,*15*_ = *6*.*74*, *p = 0*.*0203)* (Fig 10C). Thus, female offspring displayed decreased gene expression compared to male offspring. Concerning *Pparγ*, a two-way ANOVA indicated a main effect of perinatal diet *(F*_*1*,*16*_ = *4*.*9*, *p = 0*.*040)* and a sex effect *F*_*1*,*16*_ = *14*.*3*, *p = 0*.*0016*). CC female offspring showed a reduced gene expression of *Pparγ* in PAT compared to CC males (** p<0.*01)* (Fig 10D).

In conclusion, these data indicate that maternal exposure to a highly palatable food alters the gene expression of the lipogenic enzyme *Acaca* in PAT of male offspring. Moreover, sexual differences were found in this finding. PC males exhibited higher expression of *Acaca* than PC females. Additionally, sexual dimorphism was found in the gene expression of *Cpt1b* in PAT. Thus, CC females and PC females exhibited a decreased expression of this fatty acid β-oxidation enzyme when they were compared to CC males and PC males respectively. Furthermore, the offspring exposed to this diet during the perinatal period showed reduced gene expression of *Pparγ*, especially in the liver but also in PAT.

## Discussion

We have reported here the long-term consequences of the exposure to an inadequate maternal diet during the perinatal period. For the first time, we evaluated the gene expression of the most important receptors and enzymes of the endocannabinoid system after perinatal exposure to adverse nutritional conditions and the response to the CB1 blockade of feeding behavior. Furthermore, the expression of genes related to ECS, fatty acid metabolism and PPARs were also evaluated. The results strongly indicate that maternal exposure to a highly palatable food is associated to long-term alterations in the offspring of both sexes, even though these animals were weaned on standard chow diet. Specifically, we have found: a) Modifications in food preferences and in the behavioral response to a cannabinoid inverse agonist; b) Alterations in growing parameters, caloric intake, plasma metabolites and adiposity; c) Modifications on the profile of gene expression of ECS components and other metabolism-related genes; d) Sex-specific alterations. We discuss them below.

### Maternal highly palatable diet alters food choices in offspring from both sexes and modifies the response to the CB1 receptor blockade

Human and animal research has suggested that food preferences could be programmed during pregnancy and/or the lactation period [15,16,49,50]. Accordingly, we found changes in food preferences of male and female offspring from free choice dams. They preferred chocolate less during the compulsive feeding test than the control offspring, and this finding was consistent in both life periods studied (adolescence and adulthood). This decreased chocolate preference in offspring from free choice dams could indicate hypofunction in the central reward system. This state has been previously reported in animal models of chronic consumption of high fat diet from weaning and it has been associated to reduced sucrose preference [51]. However, a considerable amount of studies have reported that either maternal consumption of junk food or maternal high fat feeding increase the preference for highly palatable foods in offspring later in life, within the perinatal period [15,16,18]. In these studies, mothers preferred junk food over standard chow diet during the pregnancy and lactation [15], or mothers did not have choice and then they consumed a great amount of fat and/or sugars [16,18]. In contrast, in our study, mothers were exposed to a highly palatable diet throughout the perinatal period, including pregnancy and lactation. Curiously, we noticed that they preferred chocolate less during these periods than in pregestation. Therefore, these data suggest that the programming of food preferences during the perinatal period might depend on the volume of the inadequate diet ingested by the mother or by the mother’s preference for specific foods during the gestation and lactation periods.

The surprising modifications in food preferences of rat dams could be explained in part because these animals were exposed to a free choice between a standard chow and a highly palatable diet during a long time (eight weeks prior to mating). The palatable diet used in this experiment is hypoproteic (S1 Table). Hence, the high intake of palatable diet might not provide the protein needed during pregnancy and lactation. Consequently, rat dams could have adapted their food intake and didn’t prefer the chocolate mixture during these critical periods, so they could increase their protein intake. This hypothesis is supported by the results of previous experiments. Thus, it has been shown that malnourished rats could modify their food preferences according to their nutrient deficiency [52]. Additionally, it has been reported that craving during pregnancy may be associated to the nutritional requirements during this specific period of life [53,54].

Regarding the underlying mechanism of the food preference modifications in offspring, alterations in the opioid and dopaminergic signaling have been documented within the central reward system [16,18]. The endocannabinoid system could also play an important role in this behavior. Indeed, it keeps an important relation to the opioid and dopaminergic systems, [21,55] and it has been implicated in the intake of palatable foods [56] and/or in the perception of sweet taste [28]. In our study, we evaluated the behavioral response after an acute dose of AM251, a CB1 receptor inverse agonist. Specifically, we assessed the differential effect of AM251 on the caloric intake of both types of food (standard chow and a highly palatable diet) in the offspring from both perinatal diets. Interestingly, we noticed that animals exposed to a highly palatable diet prenatally, particularly male offspring, presented a different response. Thus, AM251 was able to decrease standard intake but not chocolate intake in male offspring from free choice dams. In contrast, AM251 decreased chocolate intake in control male offspring but not standard chow intake. Similar responses were previously reported [57]. Here, the CB1 antagonist Rimonabant decreased only standard chow intake during the withdrawal phase of an animal model of palatable diet alternation whereas any effect was found on palatable food intake during the palatable diet phase. Additionally, our data support the hypothesis that AM251 effectiveness could depend on the perception of palatability that in our study was evaluated through chocolate preference. In fact, AM251 could decrease the motivation to obtain highly palatable food when a free choice between two types of food is available [29,30,58]. According to this, male offspring from P dams in our study could perceive standard chow as more palatable, and therefore AM251 decreased more the intake of this type of food. On the other hand, female offspring exhibited a lower effect of AM251, despite the fact that they presented greater chocolate preference than males. Here, we noticed a greater tendency in female offspring from P mothers to have lower standard chow intake compared to P male offspring. However, we did not find any differences when compared to control female offspring. It is possible that a low cannabinoid activity, as a consequence of low eCB levels in the hypothalamus [36], could mask the anorexigenic effect of AM251 on chocolate preference in PC offspring. On the other hand, the estrous cycle could also influence the results due to the fact that estradiol and progesterone play an important role in food intake [59]. Moreover, it has been described as a modification in the expression of metabolism-related genes and alterations in the density and affinity of CB1 receptors according to the estrous stage [60,61]. Hence, the alterations in the receptors involved in the response of AM251 could therefore affect its effectiveness.

The differential response in AM251 and the perception of palatability could also depend on cannabinoid receptor distribution in the central reward system as we suggest above. Accordingly, previous studies have reported modifications in the gene expression of *Cnr1* after the continuous and long-term access to a highly palatable food. Then, CB1 down-regulation has been reported in the cingulate cortex, specific hypothalamic areas, hippocampus, cortex, nucleus accumbens and entopeduncular nucleus [62,63]. Additionally, lower reward sensitivity has been linked to alterations in the gene expression of *Cnr1* and *Faah* in the hippocampus together with a decreased effect of the CB1 antagonism on reducing the intake of palatable food [64]. Therefore, these data suggest that the perinatal exposure to an inadequate diet might have modified the expression of genes involved in reward permanently. Thus, offspring from palatable dams with decreased reward sensitivity and differential response to CB1 blockade could present alterations in the expression of components of ECS in the reward system, even though they were only exposed to highly palatable food perinatally.

### Maternal highly palatable food alters caloric intake, growth and metabolic parameters on offspring

In our study we observed important differences in body weight and caloric intake throughout the periods of development studied in a diet and sex-dependent manner. Thus, the P offspring were underweight at birth and were leaner in several stages of life. It is striking to note that despite the fact that the offspring were exposed to a hypercaloric diet, they were born underweight. Taking into account the low protein content of the highly palatable food used in this experiment (S1 Table), these data suggest that P offspring were exposed to a protein restriction during the perinatal period including lactation, as previously discussed in animal models exposed to cafeteria diet or junk food during these critical periods [15,16,36].

Interestingly, sexual dimorphism was found after weaning. PC males reached the same weight as controls at the end of adolescence (11^th^ postnatal week) whereas PC females did not reach it during the periods evaluated in the present study. Equally, the caloric intake relative to body weight of PC males increased during infancy, whereas caloric intake of PC female was higher during adolescence and the first part of adulthood. These data suggest that PC males displayed a delayed catch-up growth. This is a common finding in animal models of caloric and protein restriction when this encompasses both pregnancy and lactation. In these experiments, the offspring weaned on standard chow diet *ad libitum* usually exhibit hyperphagia until they reach the weight as controls [65,66]. Curiously, in our study the PC females kept lower weight during all the periods evaluated and they displayed hyperphagia during a great part of their life. Hence, this suggests that the compensatory hyperphagia was not able to modify the PC female growth and, therefore, they did not show catch-up growth.

Regarding the development of pathological phenotypes in adulthood, the delayed catch-up growth following malnutrition and underweight at birth has been considered as less deleterious when it has been compared to an early catch-up growth [65,66]. Furthermore, the prevention of the early catch-up growth has been proposed as a good strategy to prevent the debut of the features of metabolic syndrome later in life [67]. This syndrome has been defined as a combination of several risk factors that include hyperglycemia, increased blood pressure, elevated triglyceride levels, low high-density lipoprotein levels (HDL) and abdominal obesity [68]. However, in our study, PC male offspring, with a delayed catch-up growth, exhibited greater abdominal adiposity, lower HDL and increased LDL as well as hyperleptinemia compared to control male offspring. On the other hand, PC female offspring, without catch-up growth, showed a greater decrease in the levels of HDL, higher LDL and greater perigonadal adiposity than control female offspring at adulthood. Additionally, PC offspring of both sexes displayed alterations in the plasma metabolites of lipid metabolism such as higher levels of triglycerides and VLDL. These data demonstrate that PC offspring displayed signs of metabolic syndrome later in life, despite of the fact they exhibited a delayed or absent catch-up growth. Therefore, in animals perinatally exposed to a maternal cafeteria (hypercaloric and hypoproteic palatable diet) that were underweight at birth, the type of catch-up growth is not always a good predictor of the development of metabolic syndrome later in life.

According to our findings, the exposure to junk food or a high fat diet during the perinatal period has been associated to metabolic abnormalities as well, including higher adiposity in both sexes even though animals are weaned on standard chow [10–12]. In some of these studies [10,11,15], animals were born underweight and showed hyperphagia in different life stages as we have described above. Similarly, previous studies have reported alterations in leptin [10–12]. For instance, the exposure to junk food in pregnancy and lactation could lead to higher leptin expression in adipose tissue of male offspring even though animals were weaned on standard chow [12]. This finding is not surprising as leptin is involved in adipose tissue control. Indeed, in our experiments PC males exhibited the highest abdominal adiposity and, hence, they displayed higher levels of leptin as well. Additionally, previous studies have shown alteration in NEFA and/or plasma triglycerides in animals exposed to maternal high fat diet but weaned on standard chow [9,10]. Also, PC animals did not show greater plasma glucose than their respective controls. These outcomes were comparable to results previously described [12], but they differ from others [9,10]. It is important to note that in these studies the offspring were weaned on a standard chow diet. When animals are weaned on junk food or a high-fat diet, exacerbation of these metabolic abnormalities has been demonstrated [8,9,12,13]. Therefore, the significant alterations detected in the present study highlight the importance of the nutritional environment during the perinatal period, independent of the postnatal diet.

### Maternal highly palatable food modifies the profile of several genes involved in metabolism including ECS gene expression

To date, modifications in the expression of several components of the endocannabinoid system after chronic high-fat feeding or continuous access to a highly palatable food has been shown in important structures regulating metabolism such as several brain regions as we reviewed above [32,62,63,69]. To our knowledge, our study is the first that evaluates the expression of several components of ECS after maternal exposure to a highly palatable food. Previously, our team has detected alterations in hypothalamic endocannabinoids levels at birth in animals exposed to a highly palatable diet during pregestational and gestational periods [36]. Here, we have detected modifications in the ECS gene expression in the hypothalamus, liver and PAT of adult animals exposed to an inadequate maternal diet during the perinatal period.

Since the hypothalamus is a brain area involved in energy homeostasis, food intake and body weight [70], in the present study we aimed to evaluate the gene expression of ECS components in this brain structure. Regarding male offspring, we did not detect any differences in the expression of any component of the ECS including the CB1 receptor. However, it could be possible there are changes in the ECS gene expression in the hypothalamic regions implicated in the regulation of anorexigenic neuropeptides such as the arcuate nucleus [71]. Our finding is not surprising considering that these animals only showed hyperphagia during infancy and, hence, they could reach the weight as controls later in life. However, they displayed a differential response to AM251 and lower chocolate preference. Previous studies have pointed out that the reward system could be modified after hypercaloric diets without changes in the hypothalamus [51,62], although specific areas of the hypothalamus have been altered after chronic consumption of palatable foods as well [63]. After analyzing the hypothalamus, we cannot discard modifications in specific hypothalamic areas involved in food preference. On the other hand, we observed that PC females, which displayed hyperphagia until the 15^th^ postnatal week, showed an increase in the gene expression of *Cnr1* in the hypothalamus. These results are in agreement with previous studies that have shown the involvement of CB1 receptors in food intake. Thus, the deletion of a CB1 receptor has been associated to hypophagia and leanness [72,73], whereas obesity has been associated to the overexpression of neuropeptide Y (which displays orexigen properties) and higher levels of 2-AG in the hypothalamus [74]. However, in our experiments PC females remained leaner than CC females throughout the study despite presenting hyperphagia during part of their life. This apparent discrepancy could be explained by an excess of energy expenditure that could be independent of the activation of the hypothalamic CB1 receptors. Indeed, the over-expression of CB2 receptors in brain regions, including several hypothalamic nuclei, has been associated with a lean phenotype [75]. Similarly, in our experiments PC females had a great tendency to exhibit increased *Cnr2* gene expression (Fig 6B).

Moreover, we evaluated the gene expression of ECS components in the liver, another important structure regulating energy balance. Here, we found alterations in the expression of the metabolic enzymes *Napepld*, *Faah*, *Daglβ* and *Mgll* in offspring from free choice dams, without any modifications in *Cnr1*, the lipogenic enzymes *Acaca* and *Scd1*, or the β-oxidation enzyme *Cpt1a*. Specifically, PC females showed a decreased expression of *Mgll*, which is the most important 2-AG-degrading enzyme [76]. We also detected a perinatal diet effect on the gene expression of *Faah*, which is a main catabolic enzyme of anandamide that can also degrade 2-AG [77]. Therefore, the lower expression of these endocannabinoid-degrading enzymes could have led to higher endocannabinoid levels after chronic exposure to high-fat diet as it has been previously reported [78]. These findings result particularly interesting due to the fact that both, anandamide and 2-AG in the liver, decreased the gene expression of Apolipoprotein A-I, the primary protein component of HDLc, through CB1 receptor activation [79]. Hence, the decreased expression of *Mgll* and *Faah* could lead to increased endocannabinoid levels that could decrease more strongly the serum HDL in PC female offspring, as we commented previously, despite the normal expression of *Cnr1*. Although *Cnr1* was not overexpressed in our experiments, maybe as a consequence of a standard diet feeding, the increased endocannabinoid levels could reflect hyperactivation of CB1 receptors. On the other hand, the deficiency of *Mgll* has been linked to attenuation of diet-induced obesity and improvement of atherosclerosis via CB2 receptor activation [80,81]. Accordingly, we noticed that the liver of females from both perinatal groups displayed higher *Cnr2* gene expression. Therefore, it can be hypothesized that the leanness exhibited by PC females might depend on the lower MAGL expression and a putative CB2 receptor activation.

Regarding perirenal adipose tissue, we detected that PC males with hyperleptinema and higher abdominal adiposity including perirenal adiposity, exhibited reduced levels in the gene expression of *Faah* and *Daglβ* when they were compared to CC males. Considering that *Faah* is the catabolic enzyme which can degrade AEA and 2-AG, and *Daglβ* is the enzyme that synthetizes 2-AG, the data suggest that these animals could present higher anandamide levels in the white adipose tissue as it has been reported previously [82]. This could lead to an increase in lipogenesis and, therefore, fat storage (adiposity) after the stimulation of CB1 receptors [72]. Indeed, the PAT of PC males presented an increased gene expression of the *de novo* lipogenic enzyme *Acaca*. This is an important enzyme that catalyzes the synthesis of malonyl-CoA from acetyl-CoA, which finally favors the fatty acids synthesis [83]. Thus, the increased perirenal adiposity and leptinemia found in PC males is likely associated to the over-expression of *Acaca*. On the other hand, it has been found that leptin suppresses lipogenesis in the white adipose tissue via central mechanisms. In contrast, leptin cannot inhibit it when there is activation of CB1 receptors [84]. Accordingly, we noticed higher leptin levels without modifications in food intake that suggest leptin resistance and therefore an altered leptin signaling [85]. Although in PC males there were no differences in the gene expression of *Cnr1*, it is possible that an excessive activation of these receptors, maybe for increased endocannabinoid levels, could impact on the greater adiposity found. In contrast, PC females with higher perigonadal adiposity but not perirenal adiposity, exhibited greater gene expression of *Cnr1* in PAT, when compared to CC females. These results are in agreement to previous reports after chronic exposure to high-carbohydrate diets [86]. Curiously, this increment was not accompanied by other alterations in fatty acid metabolism genes and leptinemia, which suggests an isolated effect. However, female offspring from both perinatal groups, and more specifically PC females, displayed higher gene expression of *Cnr2*. Hence, one possibility that emerges from this finding is that the modifications found in *Cnr1* in female offspring could not have an impact on other metabolic outcomes as a consequence of the protective effect caused by the stimulation of the CB2 receptor. Indeed, an improvement of the obese phenotype after CB2 receptor activation has been shown in animals with diet-induced obesity [87].

On the other hand, we observed a lower gene expression of *Pparγ* in the liver and PAT of the offspring from P dams. PPARγ is a nuclear receptor that regulates the expression of lipid metabolism-related genes together with other PPARs. PPARγ plays an important role in the modulation of adipocyte hypertrophy and insulin resistance after exposure to a high-fat diet [88]. Moreover, chronic activation by cannabinoid agonists stimulates PPARγ, which is a signal of adipocyte differentiation in early stages [89]. Similarly, the down-regulation of PPARγ has been described previously in animal models after the chronic exposure to high-fat/high-sucrose diet in adipose tissue and/or liver [90,91]. Indeed, the down-regulation has been associated with altered metabolic phenotype [90]. Regarding our study, the significant down-expression of *Pparγ* in the liver of PC males suggest a reduction of fatty acid uptake in liver, which could be related to the increased levels of triglycerides in plasma and, in turn, the increased expression of the lipogenic enzyme *Acaca* in PAT. On the other hand, the decreased expression of *Pparγ* and the eCBs-degrading enzymes *Faah* and *Mgll* in the liver of PC females might indicate a hypersensitization of the PPARγ/cannabinoid receptor activity, which may be associated to an alteration of fatty acid uptake, cholesterol profile and hepatic transaminases.

### Sexual dimorphism after the exposure to a highly palatable food

The data previously discussed emphasize that the perinatal nutritional conditions differentially impact male and female offspring. Thus, when PC males were compared to control offspring, they displayed normoweight, increased abdominal adiposity, hyperleptinemia, alterations in the lipid plasmatic profile, altered response to AM251 and alteration in the expression of metabolic enzymes of the ECS and *Acaca* enzyme in perirenal adipose tissue. In contrast, when PC female offspring were compared to control female, they were underweight. They exhibited hyperphagia during part of their life, increased perigonadal adiposity but not abdominal adiposity, alterations in lipoproteins including strong reduction of HDL and higher *Cnr1* expression in hypothalamus and PAT, along with lower expression of endocannabinoid-degrading enzymes in the liver. These findings are in agreement with previous studies that have shown sex-specific profiles after the exposure to maternal junk food or high-fat diets as well [10,12–14].

Regarding the possible explanations of the sexual dimorphism documented the importance of genomic imprinting and the role of placenta during early pregnancy has been pointed out. Thus, sexual-specific alterations in DNA methylation in the placenta after high-fat feeding during pregnancy has been documented, which highlights the importance of early nutritional conditions [92]. On the other hand, the differences in gonadal hormones cannot be discarded. For instance, a previous research has documented sexual dimorphism in ECS as well, showing fluctuations in the expression of cannabinoid receptors during the estrous cycle and after gonadectomy in females [61].

In our study, the phenotype exhibited by PC females seemed more resistant to the developmental programming, even though they displayed altered *Cnr1* gene expression. However, these modifications in gene expression suggest that in harmful conditions the vulnerability of diseases could increase. For instance, the onset of menopause and the cessation of estrogens have been linked to metabolic diseases [93]. According to this, the study developed by Dahlhoff et al [13] showed that the differences between male and female offspring were less pronounced at the age of 9 months than at 5 months. Considering that the beginning of irregular cycles in female mice is around 8 months of age [94], it is possible that a reduction in estrogens could explain these differences. In our study, we realized that female offspring displayed higher gene expression of *Cnr2* at the 5^th^ postnatal month. This receptor is involved in ovulation [95], and the stimulation by a CB2 receptor agonist has demonstrated an anti-obesity effect [87]. Consequently, it could be speculated that the up-regulation of this receptor in females could exhibit a protective role against metabolic diseases until menopause.

## Conclusions

The data demonstrate that exposure to a maternal palatable diet since the preconception period predisposes animals to develop features of metabolic syndrome, and affect the feeding behavior, including the differential response to the inverse cannabinonid agonist AM251, and the expression of genes involved in cannabinoid and lipid metabolism of the offspring in a sex-specific manner, even though the animals are weaned on standard chow diet. The increased expression of *Cnr1* in the hypothalamus and PAT of PC female offspring suggest a hyposensitization of the cannabinoid activity, which may result in a reduction in chocolate preference, leptinemia and body weight at adulthood. However, the decreased expression of *Pparγ* and the eCBs-degrading enzymes *Faah* and *Mgll* in the liver of PC females suggest a hypersensitization of the PPARγ/cannabinoid receptor activity (*Cnr1* showed a tendency to decrease), which may result in an alteration of fatty acid uptake, cholesterol profile and hepatic transaminases. The decreased expression of the NAEs-degrading enzyme *Faah* and the AGs-synthesizing enzyme *Daglβ* in the PAT of PC males suggest an increase in the AEA tone and a decrease in the 2-AG tone, which may finally result in an increase in *de novo* lipogenesis (*Acaca*) in PAT and a decrease in the lipid uptake (*Pparγ*) in the liver, as well as perirenal adiposity and leptinemia. These outcomes are independent of the postnatal diet. These results emphasize the importance of nutritional conditions during these critical windows in the development of the abnormal phenotype exhibited by offspring in a sex-dependent manner. Therefore, we propose that the perinatal exposure to a highly palatable food could program the energy metabolism and feeding behavior by different mechanisms that include the affectation of the ECS. Whether the dysregulation found in the ECS could increase the vulnerability for developing diseases in presence of deleterious dietetic or hormone conditions must be addressed in future studies.

## Supporting Information

S1 Table Diet composition. (DOCX)

S2 Table Primer references for TaqMan^®^ Gene Expression Assays (Applied Biosystems). (DOCX)
