# Correction: Exposure to a Highly Caloric Palatable Diet during the Perinatal Period Affects the Expression of the Endogenous Cannabinoid System in the Brain, Liver and Adipose Tissue of Adult Rat Offspring

**DOI:** 10.1371/journal.pone.0173653

**Published:** 2017-03-06

**Authors:** María Teresa Ramírez-López, Rocio Arco, Juan Decara, Mariam Vázquez, Rosario Noemí Blanco, Francisco Alén, Juan Suárez, Raquel Gómez de Heras, Fernando Rodríguez de Fonseca

The second author’s first name is incorrect. The full name should be Rocio Arco.

The funding information incorrectly includes two grant numbers that did not support this research. The correct Funding Information should be: This work was supported by the Instituto de Salud Carlos III UEDR-FEDER, Ministerio de Economía y Competitividad UE/ERDF (PI13/0226 to F.R.F., CP12/03109 to J.S. and PSI-2012-35388 to R.G.H.), Red de TrastornosAdictivos UE/ERDF (RD12/0028/0001 to F.R.F.), CIBERobn, Consejería de Economía, Innovación y Ciencia, Junta de Andalucía, UE/ERDF (PI45403, CTS-8221, CTS-433 to F.R.F.), Consejería de Salud, Junta de Andalucía (SAS111224 to F.R.F.). Ramírez-López has been funded by a FPU predoctoral fellowship of the Spanish Ministerio de Educación, Cultura y Deporte (AP-2009-0225); J.S. holds Miguel Servet research contract from the National System of Health, ISCIII (grant numbersCP12/03109).
