# Supplementary material for: Exposure to a Highly Caloric Palatable Diet during the Perinatal Period Affects the Expression of the Endogenous Cannabinoid System in the Brain, Liver and Adipose Tissue of Adult Rat Offspring
Source: PLoS One. 2016 Nov 2;11(11):e0165432. doi: 10.1371/journal.pone.0165432 (PMC5091916; doi:10.1371/journal.pone.0165432)
Supplement: S1 Table — (DOCX) [file pone.0165432.s001.docx]

**Table S1.** Diet composition

| Diet |  | Protein |  | Carbohydrate | |  | Fat | | |  | Fiber | Sodium | Energy |
| --- | --- | --- | --- | --- | --- | --- | --- | --- | --- | --- | --- | --- | --- |
|  |  |  |  | **Overall** | **Simple carbohydrates** |  | **Overall** | **Saturated fatty acid** | **Unsaturated**  **fatty acid** |  |  |  |  |
| Standard chow |  | 16.1% |  | 60.0% | 3.3% |  | 3.1% | 22.20% | 77.70% |  | 4.0% | 0.003% | 2.9 Kcal/g. |
| Chocolate Mixture |  | 6.0% |  | 60.4% | 89.0% |  | 24.45% | 56.2% | 43.88% |  | 1.45% | 0.17% | 4.88 Kcal/g |
