# Supplementary material for: Exposure to a Highly Caloric Palatable Diet during the Perinatal Period Affects the Expression of the Endogenous Cannabinoid System in the Brain, Liver and Adipose Tissue of Adult Rat Offspring
Source: PLoS One. 2016 Nov 2;11(11):e0165432. doi: 10.1371/journal.pone.0165432 (PMC5091916; doi:10.1371/journal.pone.0165432)
Supplement: S2 Table — (DOCX) [file pone.0165432.s002.docx]

**Table S2.** Primer references for TaqMan® Gene Expression Assays (Applied Biosystems)

| **Gene description** | **Assay ID** | **Nº accession**  **GenBank** | **Amplicon Length** |
| --- | --- | --- | --- |
| ***Actb*** (Beta actin) | Rn00667869_m1 | NM_031144.2 | 91 |
| ***Cnr1*** (cannabinoid receptor 1) | Rn02758689_s1 | NM_012784.4 | 92 |
| ***Cnr2*** (cannabinoid receptor 2) | Rn03993699_s1 | NM_001164142.1 | 102 |
| ***Daglα*** (diacylglycerol lipase, alpha) | Rn01454304_m1 | NM_001005886.1 | 67 |
| ***Daglβ*** (diacylglycerol lipase, beta) | Rn01453770_m1 | NM_001107120.1 | 57 |
| ***Faah*** (fatty acid amide hydrolase) | Rn00577086_m1 | NM_024132.3 | 63 |
| ***Mgll*** (monoglyceride lipase) | Rn00593297_m1 | NM_138502.2 | 78 |
| ***Napepld*** (N-acyl phosphatidylethanolamine phospholipase D) | Rn01786262_m1 | NM_199381.1 | 71 |
| ***Acaca*** (acetyl-CoA carboxylase alpha*)* | Rn00573474_m1 | NM_022193.1 | 60 |
| ***Scd1*** (stearoyl-Coenzyme A desaturase 1) | Rn00594894_g1 | NM_139192.2 | 86 |
| ***Cpt1a*** (carnitine palmitoyltransferase 1a, liver) | Rn00580702_m1 | NM_031559.2 | 64 |
| ***Cpt1b*** (carnitine palmitoyltransferase 1b, muscle) | Rn00682395_m1 | NC_005106.4 | 83 |
| ***Pparα*** (peroxisome proliferator activated receptor alpha*)* | Rn00566193_m1 | NM_013196.1 | 98 |
| ***Pparγ*** (peroxisome proliferator activated receptor gamma) | Rn00440945_m1 | NC_005103.4 | 105 |
